# *Notes from the Field:* Clade II Mpox Surveillance Update — United States, October 2023–April 2024

**DOI:** 10.15585/mmwr.mm7320a4

**Published:** 2024-05-23

**Authors:** Alexandra Tuttle, Christine M. Hughes, Mitchell Dvorak, Leah Aeschleman, Whitni Davidson, Kimberly Wilkins, Crystal Gigante, Panayampalli S. Satheshkumar, Agam K. Rao, Faisal S. Minhaj, Bryan E. Christensen, Jennifer H. McQuiston, Christina L. Hutson, Andrea M. McCollum

**Affiliations:** ^1^Division of High-Consequence Pathogens and Pathology, National Center for Emerging and Zoonotic Infectious Diseases, CDC; ^2^Chenega Enterprise Systems and Solution, LLC, Chesapeake, Virginia.

**Summary**

**What is already known about this topic?**

Since the global mpox outbreak began in 2022, mpox cases have continued to occur in the United States.

**What is added by this report?**

After the peak of the 2022 mpox outbreak, when approximately 3,000 cases per week were reported, cases declined sharply and remain significantly lower (approximately 59 reported cases per week during October 1, 2023–April 30, 2024). Most new mpox cases occur in unvaccinated persons.

**What are the implications for public health practice?**

CDC recommends that persons at risk for mpox exposure, who have not previously recovered from mpox (including certain gay, bisexual, and other men who have sex with men) complete the 2-dose JYNNEOS vaccination series.

Two clades of monkeypox virus (MPXV) are known to cause human illness: clade I, which is endemic in Central Africa and is currently increasing in the Democratic Republic of the Congo, and clade II, which caused a global outbreak starting in 2022. Clade II–associated disease is considered less severe than that of clade I and is typically self-limiting; however, immunocompromised persons, especially those with advanced HIV (i.e., CD4 T lymphocyte cell count <200 cells/mm^3^), have experienced more severe infections (*1**,**2*). Clade II MPXV continues to circulate at low levels in the United States, but no cases of clade I MPXV have been reported. National mpox case counts peaked at approximately 3,000 per week during late July–August 2022 (Figure), then sharply declined and remain substantially lower than case counts during the peak (59 cases per week during October 1, 2023–April 30, 2024). This report summarizes mpox surveillance data reported to CDC during October 1, 2023–April 30, 2024. This activity was reviewed by CDC, deemed not research, and was conducted consistent with federal law and CDC policy.*

**FIGURE:**
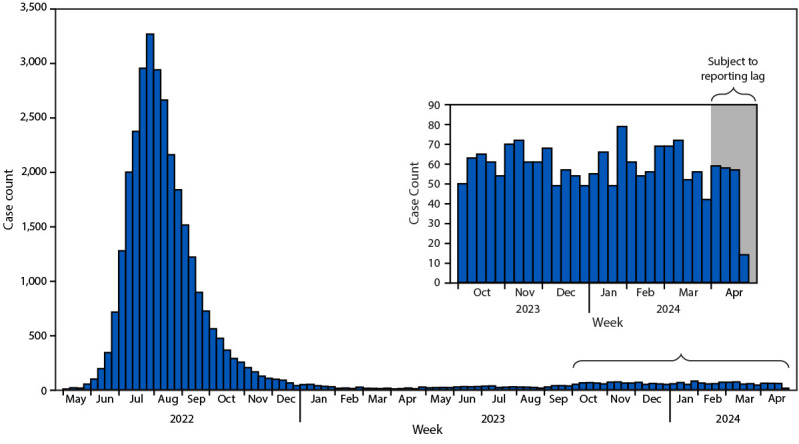
Clade II mpox cases (probable* and confirmed†), by epidemiologic week — United States, May 2022–April 2024^§^ **Abbreviations:** IgM = immunoglobulin M; MPXV = monkeypox virus; PCR = polymerase chain reaction. *Probable cases are defined as infections in persons with no suspicion of other recent *Orthopoxvirus* exposure (e.g., Vaccinia virus in ACAM2000 vaccination) and demonstration of the presence of at least one of the following criteria: 1) *Orthopoxvirus* DNA by PCR testing of a clinical specimen, 2) *Orthopoxvirus* using immunohistochemical or electron microscopy testing methods, or 3) demonstration of detectable levels of antiorthopoxvirus IgM antibody during the 4–56 days after rash onset. ^†^ Confirmed cases are defined as demonstrated presence of MPXV DNA by PCR testing or next-generation sequencing of a clinical specimen or isolation or MPXV in culture from a clinical specimen. ^§ ^Data on confirmed and probable mpox cases collected by jurisdictional public health departments and electronically reported through the National Notifiable Disease Surveillance System or via a standardized case report form.

## Investigation and Outcomes

During October 1, 2023–April 30, 2024, a total of 1,802 probable and confirmed^†^ mpox cases were reported to CDC by 42 states, the District of Columbia, and Puerto Rico. Whereas local mpox case counts have fluctuated, national counts have remained steady, with an average of 59 cases per week. Cases continue to occur primarily among cisgender men (1,054 [94%] of 1,121 who reported data on gender identity) and those who identified as gay or bisexual (326 [90%] of 361 who reported sexual orientation data). Most cases (62%) occurred among persons aged 25–40 years, with a median age of 34 years (range = 0–76 years); six (0.4%) cases occurred among persons aged <18 years. Race and ethnicity were reported for 1,651 (98%) cases; among these persons, 526 (34%) identified as Hispanic or Latino (Hispanic), 535 (32%) as White, 410 (25%) as Black or African American (Black), 54 (3%) as Asian, 31 (2%) as multiracial, and 59 (4%) as another race, including American Indian or Alaska Native and Native Hawaiian or Pacific Islander.^§^ Among 593 persons with mpox who reported HIV status, 282 (48%) were HIV-positive. Among 1,429 patients with mpox and with hospitalization data reported, 145 (10%) were hospitalized during their illness; among these, 72 reported HIV status, 49 (68%) of whom were HIV-positive. Since October 2023, five patients with mpox have died. Among 684 (38%) persons with mpox who reported vaccination status, 458 (67%) persons reported no vaccination against mpox, and 226 (33%) had received at least 1 dose of vaccine against mpox or smallpox. Of those receiving at least 1 dose, only two (1%) were hospitalized during their illness.

## Preliminary Conclusions and Actions

MPXV transmission continues at low levels in the United States. CDC continues to perform genomic sequencing and MPXV clade–specific testing to identify MPXV mutations that affect medical countermeasure effectiveness (i.e., resistance to the antiviral tecovirimat) and to aid in clade I surveillance. To date, no clade I mpox cases have been detected in the United States.

The current average of 59 reported cases per week represents a fifty-five-fold reduction, compared with the peak of 3,274 cases reported during the week beginning July 31, 2022 (the peak outbreak week); levels have remained stable since October 2023. Compared with cases reported during May 10, 2022–September 30, 2023, the proportion of cases among Black persons declined by 7 percentage points (from 32% to 25%) and increased among Hispanic persons by 3 percentage points (from 31% to 34%) since October 2023.^¶^ Hospitalizations during this period have increased slightly (10% of cases during October 1, 2023–April 30, 2024, compared with 8% during May 10, 2022–September 30, 2023). Mpox-related deaths in the United States remain rare (0.3% of cases since October 2023). More than two thirds (67%) of new mpox cases occur among persons not previously vaccinated. Since the start of the outbreak, 39% of persons at risk for mpox exposure have received at least 1 dose of vaccine, and 25% have received 2 doses (*3*). Thus, the majority of persons at risk for mpox exposure remain unvaccinated. CDC recommends that persons at risk for mpox exposure, who have not previously recovered from mpox, receive 2 doses of JYNNEOS vaccine and complete the 2-dose vaccination series, irrespective of time since initial dose or route of vaccination.**^,^^††^

## Footnotes

* 45 C.F.R. part 46, 21 C.F.R. part 56; 42 U.S.C. Sect. 241(d); 5 U.S.C. Sect. 552a; 44 U.S.C. Sect. 3501 et seq.

† https://www.cdc.gov/poxvirus/mpox/clinicians/case-definition.html (Accessed April 24, 2024).

§ Persons of Hispanic or Latino (Hispanic) origin might be of any race but are categorized as Hispanic; all racial groups are non-Hispanic.**
**

¶
https://www.cdc.gov/poxvirus/mpox/response/2022/demographics.html

** https://www.cdc.gov/vaccines/hcp/acip-recs/vacc-specific/smallpox.html (Accessed April 24, 2024).

†† https://www.cdc.gov/poxvirus/mpox/vaccines/vaccine-recommendations.html (Accessed May 9, 2024).
